# Rational Engineering of a Human Anti-Dengue Antibody through Experimentally Validated Computational Docking

**DOI:** 10.1371/journal.pone.0055561

**Published:** 2013-02-06

**Authors:** Luca Simonelli, Mattia Pedotti, Martina Beltramello, Elsa Livoti, Luigi Calzolai, Federica Sallusto, Antonio Lanzavecchia, Luca Varani

**Affiliations:** 1 Institute for Research in Biomedicine, Bellinzona, Switzerland; 2 Institute for Health and Consumer Protection, Joint Research Centre, Ispra, Italy; 3 Institute of Microbiology, Eidgenössische Technische Hochschule (ETH) Zürich, Zürich, Switzerland; University of Texas Medical Branch, United States of America

## Abstract

Antibodies play an increasing pivotal role in both basic research and the biopharmaceutical sector, therefore technology for characterizing and improving their properties through rational engineering is desirable. This is a difficult task thought to require high-resolution x-ray structures, which are not always available. We, instead, use a combination of solution NMR epitope mapping and computational docking to investigate the structure of a human antibody in complex with the four Dengue virus serotypes. Analysis of the resulting models allows us to design several antibody mutants altering its properties in a predictable manner, changing its binding selectivity and ultimately improving its ability to neutralize the virus by up to 40 fold. The successful rational design of antibody mutants is a testament to the accuracy achievable by combining experimental NMR epitope mapping with computational docking and to the possibility of applying it to study antibody/pathogen interactions.

## Introduction

Improving our understanding of the structural rules governing antibody/antigen interactions is expected, in the long run, to accelerate vaccine development, since most modern vaccines aim to elicit an antibody response, and to help us design better antibodies for passive immunization or biotechnology applications such as the production of bio-recognition elements for target detection. As a proof of concept, we set forth to structurally characterize the binding of one antibody to the four existing Dengue Virus (DenV) serotypes and use this information to rationally alter its immunological properties, eliminating cross-reactivity and improving its ability to neutralize the virus.

DenV is responsible for 20,000 deaths and 500,000 hospitalizations annually [1], with economic impact rivaling that of malaria. Its epidemic activity and geographic expansion are increasing as climate changes, travel and urbanization create favorable conditions for the mosquito spreading it [2]. No cure or vaccine is currently available, mostly due to the presence of four serotypes and to a poorly understood process called Antibody Dependent Enhancement, where antibodies raised against a previous Dengue infection facilitate subsequent infection by another serotype [3]. In addition to its biomedical importance, the presence of related serotypes and the fact that they are structurally well characterized both at the protein and viral capsid level make DenV a good model for the study of antibody/antigen interactions. Although structural studies often concentrate on the complex between an antibody and a single serotype, usually the one against which the antibody is most effective, a comparison of the same antibody bound to antigens that it can and cannot neutralize may, in fact, teach us why it is only effective against some of them.

Having isolated a panel of human monoclonal antibodies from a donor recovered from infection from Dengue Virus serotype 2 (DenV2) [4], we selected and characterized one that would: *i*) bind all four DenV serotypes; *ii*) effectively neutralize only some of them and *iii*) bind to the so-called DIII, a small ig-like domain part of the E protein, whose homodimers are the main component of the viral surface [5], [6], [7] and a dominant target for the human antibody response against DenV [4], [8], [9], [10].

We previously characterized the interaction between DV32.6, an antibody with the above mentioned properties, and DenV4 [11]. This alone however, cannot explain why DV32.6 can neutralize the other three serotypes given that the antibody binds stronger to its epitope on DenV4 rather than DenV1 or DenV3. If the antibody/antigen interaction were identical in all serotypes, then the antibody should fail to neutralize DenV1 and DenV3 just as it fails to neutralize DenV4.

Here we aim to elucidate the structure of DV32.6 in complex with all the remaining DenV serotypes and exploit the differences to rationally design mutated antibodies with i) selectively altered binding specificity and ii) improved ability to neutralize the virus.

We first use NMR epitope mapping to define the binding site of DV32.6 on DIII of all four DenV serotypes. We then use this information to filter computational predictions of the antibody/antigen complexes. Analysis of the resulting three dimensional structures proved sufficiently accurate for the rational design of antibody mutants with selectively altered binding specificity or improved neutralization properties.

## Results

### Antibody DV32.6 Binds to All Dengue Serotypes

**Figure 1:**
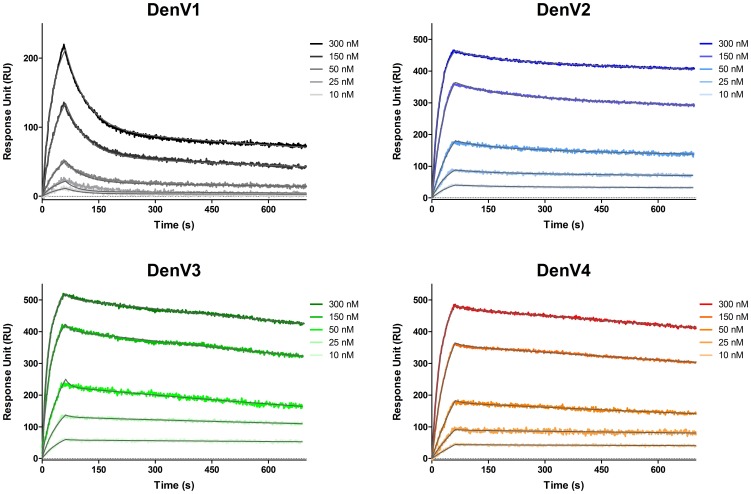
Antibody DV32.6 binds the four Dengue serotypes with different affinity. SPR sensorgrams showing association and dissociation of DV32.6 to DIII of each Dengue serotype. The antibody was immobilized on the sensor surface, followed by injection of DIII at the concentrations indicated in the figure. The line fitted to the experimental data and used to calculate the binding affinities is drawn in gray. KD values are 145±9 nM for DenV1; 7±0.2 nM for DenV2; 73±16 nM for DenV3; 34±7 nM for DenV4. Despite binding more weakly to DenV1 and DenV3 than DenV4, the antibody neutralizes those serotypes better than DenV4.

DV32.6 is part of a panel of human monoclonal antibodies isolated from a donor recovered from infection by DenV2 [4]. It binds to DIII of all four DenV serotypes with KD 145±9 nM for DenV1; 7±0.2 nM for DenV2; 73±16 nM for DenV3; 34±7 nM for DenV4 according to SPR (Figure 1). The ability of DV32.6 to neutralize the virus was assessed by flow cytometry assays measuring the number of cells infected by DenV vaccine strains in the presence of different amounts of antibody. There is no direct correlation between DIII binding affinity and neutralization: the antibody is more efficient at neutralizing DenV2, DenV1 and DenV3 despite binding more strongly to DIII of DenV4 (Figure S1). Association and dissociation rates show no obvious correlation to the neutralizing activity, either. The approximate concentration of antibody required to neutralize 50% of the viral activity is 2 µg/ml for DenV2, 3 µg/ml for DenV3, 4 µg/ml for DenV1 and >74 µg/ml for DenV4. Incidentally, DV32.6 was isolated from a patient recovered from DenV2 and is most effective against this very serotype. Binding assays on isolated DIII allow us to compare the binding affinity of the antibody for its epitope but the natural target for the antibody is the full virus, where the surface of DIII is partially covered by neighbouring protein domains. In contrast to DIII binding, ELISA performed at 37°C on the full virus show that binding to DenV4 is not stronger than to other serotypes (Figure S1). No binding curve is obtained, instead, when performing ELISA on the full virus at 4°C; this will be later discussed in the context of the structural data.

### NMR Epitope Mapping

Solution NMR spectroscopy was used to characterize, at the residue level, the epitope (i.e. the region of the antigen that interacts with the antibody) of DV32.6 on DIII from each DenV serotype. In a so-called ^15^N-HSQC experiment, one of the simplest NMR experiments, each protein residue generates an individual signal. The position of these signals is sensitive to the local chemical environment, so much so that ^15^N-HSQCs are considered protein fingerprints. We exploit this property to determine the epitope: when DV32.6 binds to DIII, interface residues experience a different chemical environment and their NMR signal changes as a consequence. By comparing the NMR spectrum of DIII free or in complex with the antibody we can identify which signals change. Knowing the assignments, i.e. determining which signal belongs to which antigen residue, we can therefore determine the residues affected by antibody binding (Figure S2 and S3). NMR assignments are publicly available for DIII of three serotypes [12], [13], [14]. We assigned DIII of DenV3 according to standard techniques. We utilize a purely qualitative approach: if a signal changes position or disappears, then we know that the residue generating such signal is affected by antibody binding. In our experience, a quantitative analysis of the changes is neither required nor satisfactory in the case of large and poorly behaved complexes with low spectral quality. Nonetheless, the accuracy attainable by qualitative analysis even in such complicated cases is sufficient, as we have previously shown for a TCR/pMHC complex (similar in many ways to our DIII/DV32.6 complexes) that was subsequently validated by an x-ray structure [15] and in an RNA/protein complex later verified by an NMR structure [16]. Furthermore, by relying on simple and sensitive ^15^N-HSQC experiments, or their TROSY equivalent, we can adopt this approach even for the most difficult cases when more sophisticated NMR approaches are not suitable. Such NMR experiments may theoretically offer more information, but they fail to do so due to lack of sensitivity in the case of our DIII/antibody complexes.

**Figure 2:**
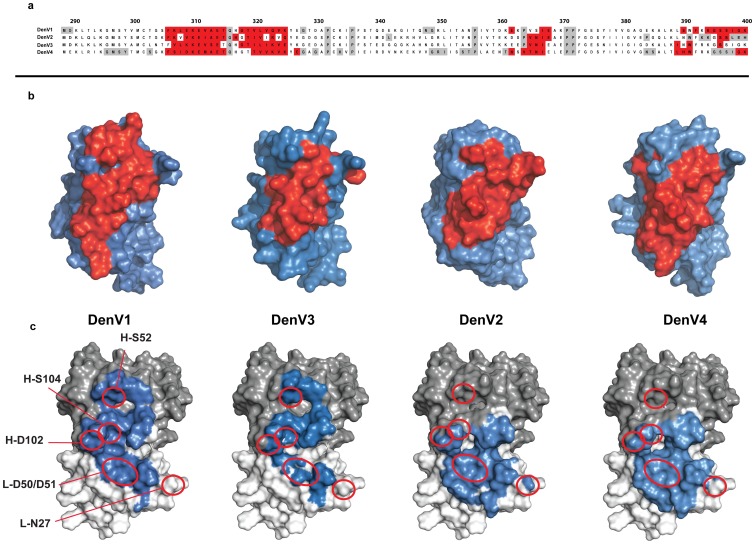
Antibody DV32.6 interaction with the four Dengue serotypes. NMR epitope mapping results (a, b): residues whose NMR signal is affected by antibody binding are indicated in red on the sequence (a) and on the surface representation (b) of DIII of each DenV serotype. Residues for which no NMR information is available are coloured gray in the sequence. The discontinuous epitope shows slight variations amongst serotypes both in sequence and structure, including some conserved residues and others that are not. The former explain why DV32.6 can bind to all four serotypes, the latter are likely responsible for the different binding and neutralization properties. Computational docking results (c). Surface representation of the antigen binding site of DV32.6. Antibody residues predicted to interact with DenV are shown in blue; light and heavy chains are in light and dark gray, respectively. Both antibody chains contribute to the binding interface in DenV1 and DenV3 whereas only the light chain and the H3 loop bind to DenV2 and DenV4. Some of the residues mutated to alter the antibody properties in a predictable way (see text) are indicated by red circles and labelled on DenV1.

The NMR signal of approximately 20% of the surface residues of DIII is perturbed upon binding of DV32.6. No unassigned peak shows chemical shift changes upon complex formation with the exception of one signal that presumably belongs to Q316, in the middle of the epitope. The epitope centers around residues 306–325 and the antibody footprint shows only slight variation amongst serotypes (Figure 2a and 2b). It includes residues conserved in all serotypes, explaining why DV32.6 binds all four of them, and residues that are not conserved, which are probably responsible for the different binding and neutralization properties. However, sequence or structural analysis of the epitope offers no information on the antibody residues important for interaction. To further characterize the binding interface we thus predicted the three-dimensional structure of the complex between DV32.6 and DIII by computational docking, guided and validated by the NMR epitope mapping data.

### Experimentally Validated Computational Docking

Computational docking predicts the structure of a multi-molecular complex starting from the separated structures of the individual components. Experimental structures of DIII from each serotype are available [6], [13], [14], [17] and were used for docking. We predicted the structure of antibody DV32.6 by homology modeling according to the canonical structure method [18]. Although docking a model, thus making a model of a model, is known to generate problems in protein-protein docking, we and others have clearly shown that this is not the case for antibodies [11], [18], since they can be modeled with high accuracy and precision in the vast majority of cases and exceptions are readily recognized [19]. Briefly, antibody antigen binding loops can adopt a limited set of conformations constrained by loop length and presence of specific amino acids at key positions [20]. Exploiting these constraints yields highly reliable antibody models, except for the H3 loop that can adopt highly variable conformations. We thus generated multiple models of DV32.6 [11], differing mainly in the conformation of the H3 and L3 loop and in the relative position of the six antigen binding loops. In particular, the H3 loop extends outwards in some of our models but presents a rather flat interacting surface in others. The purpose is two-fold: on one hand by using multiple models we increase the chances that at least one of them is accurate; on the other hand the ensemble of conformations may simulate the flexibility available to long protein loops such as the H3 of DV32.6. We then docked each antibody model independently to DIII with the program RosettaDock [21] and validated the results as described in the methods section. The difference between our approach and “ensemble docking” [22] protocols is that in the latter docks an ensemble of multiple conformations and uses a scoring function to select the best model. We, on the other hand, evaluate the docking of each conformation according to its agreement with the experimental data. We have previously shown that combining NMR mapping and docking in this way significantly increases the accuracy of computational docking [11]. In fact, the initial docking search we performed failed to generate a so-called “scoring funnel”, an ensemble of similar conformations significantly more accurate than the others according to the docking algorithm. Without our experimentally validated approach we would not be able to discriminate amongst a dozen or so solutions recognized as equally valid by the computational algorithm alone. Further refinement of the structure in better agreement with the NMR data (see methods) would often generate two scoring funnels, only one of which was in agreement with the experimental data.

### Interaction of Antibody DV32.6 with the Four Dengue Serotypes

According to our NMR validated docking predictions, DV32.6 primarily recognizes DIII residues located on adjacent beta strands, covering between 25 and 28 residues with a buried surface area between 684 Å^2^ (DenV2) and 768 Å^2^ (DenV1). These values are in line with those obtained from x-ray structures of other antibodies against DIII. Antibody 2H12 has buried surface area between 491 Å^2^ (DenV4) and 652 Å^2^ (DenV1). Antibody 1A1D-2 has a larger buried surface of 843 Å^2^ and antibody 4E11 buries between 758 Å^2^ (DenV3) and 894 Å^2^ (DenV1) [23], [24], [25]. The predicted interface of antibody DV32.6 is dominated by electrostatic interactions and features several intermolecular hydrogen bonds and salt bridges. All DIII residues predicted to be at the interface by the selected docking solutions are also affected by complex formation in the NMR epitope mapping experiment.

K310, conserved in all serotypes, is at the center of the interface and involved in multiple intermolecular contacts. The epitope has several non-conserved residues, which are probably responsible for the different binding and neutralization properties of DV32.6. Indeed, residues 307, 309, 323, 325 and 327 differ among serotypes and appear to be involved in antibody binding. Residue 309, for instance, is Glu, Val, Lys or Asp in DenV1 to DenV4, respectively, and it may not be surprising that it has a different effect on the antibody partner. Perhaps surprisingly, some conserved DIII residues appear to have different antibody partners in different serotypes: E311, for instance, interacts with either the backbone of H-D102 (D102 of the antibody Heavy chain), L-S32 (S32 in the antibody Light chain) or L-S93. This may suggest conformational plasticity or may very well reflect a limit in the precision of the computational models. Even if the specific interactions are wrong, however, the models provide an indication of which residues are involved in intermolecular contacts. We may not know if E311 interacts with H-D102, L-S32 or L-S93, for instance, but we know that it is involved in electrostatic interactions with the antibody. This level of information was sufficient to successfully design antibody mutants.

Analyzing the predicted antibody interface (Figure 2c) shows differences that could be exploited to selectively alter the binding properties: the light chain (L1, L2, L3) and H3 antigen binding loops interact with every serotype; the H2 loop interacts only with DenV1 and DenV3 and the H1 loop has no contact with any serotype. Although the total interface area is similar, the light and heavy chains equally contribute to it in DenV1 and DenV3, whereas 80% of the interface is formed by the light chain in DenV2 and DenV4.

### Rational Antibody Engineering to Selectively Alter its Binding Properties

By analyzing the three-dimensional models of the antibody/DIII complexes we designed several antibody mutants with the intent of further verifying our computational predictions, altering the binding properties of the antibody and ultimately improving its ability to neutralize the virus.

**Figure 3:**
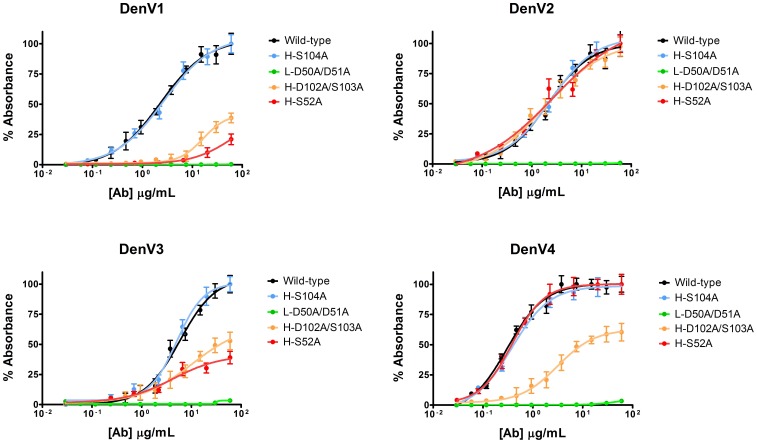
Rational antibody engineering to selectively alter the binding specificity. Binding assays (ELISA) of representative mutants for the four DenV serotypes are shown; higher y values correspond to increased antibody binding to DIII. The structural position of these mutants is indicated in Figure 2c. Wild-type DV32.6 (black) binds to all serotypes and H-S104A does not alter its properties. The L-D50A/D51A mutant cannot bind to any serotype. The H-D102A/S103A mutant binds to DenV2 and has severely limited binding to the other serotypes. The H-S52A mutant can only bind to DenV2 and DenV4.

As a first test we aimed to modify the antibody without affecting its binding to DIII, proving that we can identify and avoid critical residues. Antibody sequence analysis can easily predict which residues belong to antigen binding loops and may, therefore, interact with the antigen. Our models go a step further and can identify which of these residues are not directly involved in intermolecular contacts and can thus be mutated without adverse consequences. We thus selected and mutated a subset of such residues in each of the three heavy chain antigen binding loops. As predicted, all the following mutations in the antigen binding loops didn’t alter the antibody binding properties: H-S104A (Figure 3); H-T31A; H-S54Q; H-S103V; H-T106A. Binding assays for all the mutants described in the manuscript are included as figures S4 and S5.

To further validate our models we then aimed to abolish antibody binding to all serotypes. Residues L-D50 and L-D51 are predicted to be at the center of the antibody/DenV interface, forming an intermolecular network of hydrogen bonds and salt bridges in all serotypes. If our models are correct, disrupting this network should have a profound effect on the interface. Indeed, the double mutant L-D50A/D51A completely abolishes antibody binding (Figure 3). The same result is obtained when mutating the nearby H-S105D, since the negative charge introduced interferes with the negatively charged sidechain of L-D50.

Having shown that our approach can identify critical interface residues, we tackled the much more difficult task of improving the antibody properties. Generally speaking, increasing antibody selectivity is a useful exercise to eliminate cross-reactivity with undesired antigens or to design bio-recognition elements for specific antigen subtypes. As a proof of concept, we altered the interaction between DV32.6 and DenV, obtaining an antibody mutant specific for DenV2 and another that binds only DenV2 and DenV4.

According to our models, residues H-D102/S103, belonging to the H3 antigen binding loop, point away from the DenV2 antigen. Therefore, mutating them should have no effect on this serotype. They are, instead, in close proximity of DenV4 and DenV3. Finally, H-D102 is predicted to form an intermolecular salt bridge in DenV1. In agreement with this prediction, the H-D102A/S103A mutant binds DenV2 like the wild-type antibody but it has severely reduced binding to all other serotypes (Figure 3).

Since the antibody H2 loop is predicted to interact with DenV1 and DenV3 but not DenV2 and DenV4, we designed the H-S52A mutant to prevent antibody binding to DenV1 and DenV3 while leaving the other two serotypes unaltered (Figure 3). This mutant agrees with the computational prediction that the antibody uses different binding modes to interact with the serotypes as illustrated in Figure 2c.

### Rational Antibody Engineering to Improve the Neutralization Properties

**Figure 4:**
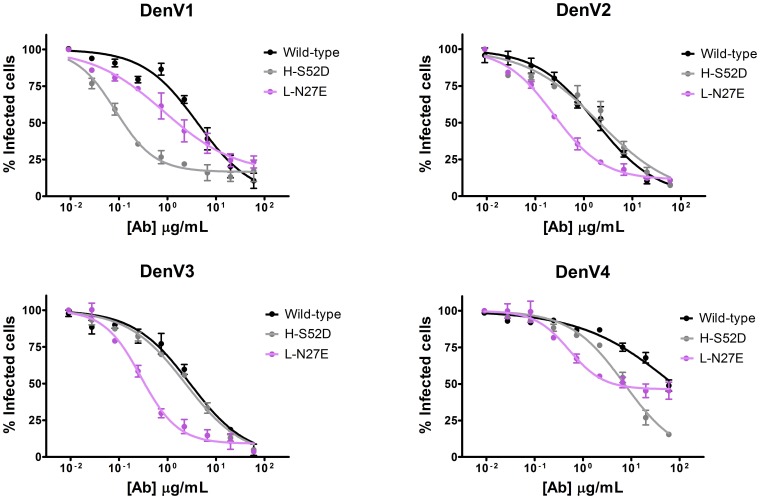
Rational antibody engineering for improved virus neutralization properties. We designed two antibody mutants with the intent of improving its neutralization properties. H-S52D (gray) neutralizes DenV1 40 times more efficiently than the wild-type (black) and L-N27E (violet) is better than the wild-type in all serotypes, albeit to a lesser extent. Viral neutralization assays are shown; the amount of infected cells (y axis) decreases at increasing antibody concentration (x axis). In comparison to the wild-type, a lower concentration of mutants is required to neutralize the same amount of virus.

The aforementioned mutations are a testimony to our ability to rationally alter the antibody binding properties and specificity. Improving the ability of therapeutic products to protect from infection is, however, the main goal of antibody engineering. As a proof of concept, analysis of the predicted antibody/antigen interfaces allowed us to design a mutated antibody 40 times more efficient than the wild-type in neutralizing DenV1 and another mutant more efficient against all serotypes, albeit to a lesser extent (Figure 4).

**Figure 5:**
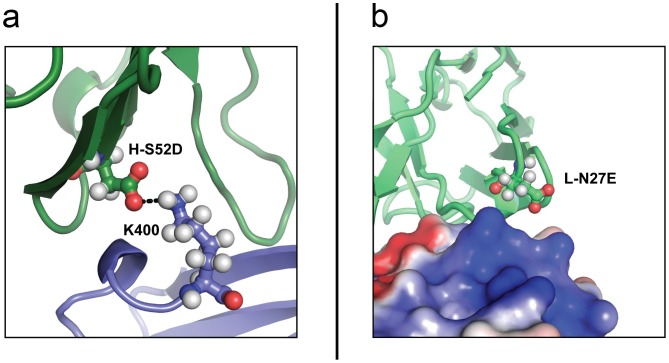
Rational antibody engineering, a structural view. a) H-S52 is close to the positive sidechain of K400 in DenV1. Mutating it to the negatively charged H-S52D favours the interaction and this mutant is 40 fold more effective than the wild type at neutralizing DenV1. The antibody is shown in green and Denv1 in blue. b) DV32.6 is shown as green cartoon over the electrostatic surface of DenV3; positively charged surfaces are in blue and negatively charged surfaces in red. L-N27 is close to a positive patch conserved in all serotypes. Introducing a negative charge (L-N27E) favours the interaction, resulting in improved neutralization properties. The L-N27E substitution was preferred to L-N27D since the longer sidechain was thought to bring the charged antibody moiety closer to DIII.

Our models show that H-S52 is in close proximity to a positively charged lysine sidechain in DenV1 (Figure 5). Introducing a nearby negative charge should favor the formation of intermolecular salt bridges, possibly resulting in improved neutralization properties. Indeed, the H-S52D antibody is 40 times more efficient in neutralizing DenV1; the estimated antibody concentration required to achieve 50% neutralization (EC_50_) is 0.1 µg/ml for HS52D and 4.1 µg/ml for wild-type DV32.6 (Figure 4). SPR indicates that the k_off_ is identical to the wild-type while the k_on_ improves from 0.95±0.01×10^−4^ to 4.8±0.4×10^−4^ nM^−1^s^−1^, resulting in a KD of 28±2 nM versus 145±9 nM. DenV4, where H-S52D is also a slightly better neutralizer than the wild-type, has a similar improvement in k_on_ (1.5±0.1×10^−4^ for the wild-type, 5.3±1.0×10^−4^ nM^−1^s^−1^ for H-S52D) and the k_off_ improves as well (from 5.1±1.3×10^−3^ to 0.47±0.1×10^−3^ s^−1^) resulting in KD of 0.9±0.04 nM for the mutant versus 34±7 nM for the original antibody.

L-N27, instead, is predicted to be close to a positively charged region in all serotypes (Figure 5) so introducing a negative charge should favor the interaction with all of them. An N>E rather than N>D mutation was introduced because a longer side chain would get closer to the antigen without creating clashes, according to the models. Indeed, the L-N27E mutant has improved neutralization properties for all serotypes (Figure 4). The L-N27E mutant is 3 times more effective on DenV1, 6 on DenV2, 9 on DenV3 and 17 on DenV4 (ratios between the EC_50_ for wild-type and mutant antibody). KD is 5 times stronger for DenV1, 23 times stronger for DenV2, 4 times for DenV3 and 38 times for DenV4. The mutant has improved K_on_ in all serotypes whereas the K_off_ is equal to the wild type for DenV1 and DenV3. Unfortunately, the effects of the H-S52D and L-N27E mutations are not additive; the double mutant shows better neutralization only for DenV3. Apparently, the interaction cannot tolerate the simultaneous introduction of two negative charges on the other serotypes.

## Discussion

Strategies for optimizing and improving antibody properties are highly desirable, either to increase their efficacy or to alter their binding specificity. Generally speaking, abolishing or altering antibody binding to undesired antigens might confer selectivity to an otherwise broadly reactive antibody, with practical uses in avoiding cross-reactivity or designing specific biomarkers (or biosensors). On the other hand, antibodies that can recognize only selected serotypes on variable pathogens like Influenza or Dengue may be engineered to become more broadly reactive. Antibody optimization can be achieved by randomizing its sequence with display technologies [26] or by structural analysis and rational modification of the antibody/antigen interface. This latter strategy is often neglected and generally thought to require high resolution crystallographic structures [27], which are not always available. As a proof of concept, we here show that structure-based antibody engineering is feasible even without x-ray information and can be achieved by computational methods guided and validated by a limited set of rapidly obtained experimental results. We have previously shown that NMR mapping can provide an accurate representation of protein-protein interfaces [15] and that this can greatly increase the accuracy of computational docking by validating its results [11]. Here we move further ahead by showing that computational models of antibody/antigen complexes allowed us to rationally design antibody mutants that improve its properties by *i*) disrupting binding only to selected serotypes and *ii*) improving virus neutralization up to 40 fold.

Antibody DV32.6, isolated from the serum of a human donor recovered from DenV2 infection, can efficiently neutralize three of the four DenV serotypes but binds to all four in the so-called DIII, the most variable domain of the protein forming the viral surface and the target of many potent antibodies described so far [4], [23]. We identified the epitope of DV32.6 on each serotype at the residue level with solution NMR spectroscopy and used this information to guide and validate computational docking simulations yielding three-dimensional models of the antibody/antigen complexes. Visual analysis of these NMR validated computational structures allowed us to design a total of 22 antibody mutants, only 4 of which did not have the predicted effect: a testament to the accuracy of our approach that is unlikely to arise from a random process. The mutants that did not behave as desired involved: disruption of a predicted hydrogen bond that did not result in a detectable loss of binding; a Ser to Gly substitution that presumably altered the antibody structure and led to total loss of binding; double substitutions that did not yield the cumulative effect of the single mutants.

The Rosetta suite is probably the most successful software for protein folding and engineering [28]. We thus utilized it to complement our visual structural analysis. In particular, we attempted to design antibody mutants that would increase the binding affinity with the RosettaDesign [29] software. However, the algorithm is biased towards the introduction of hydrophobic residues at the intermolecular interface and fails to identify the electrostatic mutations that yield increased virus neutralization in our case. Unfortunately, we were not able to generate and test the hydrophobic mutations suggested by RosettaDesign. Hydrophobic antibodies are often problematic due to aggregation and lack of specificity (“sticky antibodies”); moreover, their refolding from inclusion bodies, if produced in *E.Coli*, tends to be very difficult.

Automated mutant design by the Rosetta software does not identify the electrostatic mutations that improve the antibody properties. Given the mutations, however, we wondered if a docking algorithm would be able to provide a confirmation of their efficacy. Judging this is not straightforward since a simple energetic comparison between wt and mutant by the empiric Rosetta scoring function is unlikely to provide meaningful results. We, thus, decided to look for the presence of so-called “scoring funnels”. These are generated when the docking algorithm finds a large number of similar structures with a pseudo-energy score significantly better than the other structures. This results in a funnel shape in a plot of docking score versus structural similarity (RMSD from a reference structure). Scoring funnels are often considered an indication of an accurate docking solution, although exceptions are not uncommon, and in our experience are more likely to happen when the binding affinity between docking partners is high. In the case of the DV32.6 mutants there is no scoring funnel, nor we could detect any other indicator that would distinguish an effective mutation (e.g. L-N27E, which increases antibody binding) from an ineffective one (e.g. L-D50A/D51A, which abolishes binding). On the contrary, we had a scoring funnel only when docking the L-D50A/D51A mutant.

Software for protein design has leaped forward in recent years but it is not yet completely reliable. The advantage of human visual structural analysis, we believe, is that it can detect plausible intermolecular interactions those requirements may not be directly satisfied by the model but could be met with limited structural alterations. In an oversimplified example, an algorithm may suggest a mutation to form an intermolecular hydrogen bond if the two required chemical groups are within 3.5 Å; if the distance is 4.5 Å, however, it would fail to recognize the possibility. Human analysis, on the other hand, might recognize that a relatively minor structural rearrangement would bring the two chemical groups within bonding distance. This rearrangement might happen because proteins are flexible or, more simply, because the position of the chemical groups cannot be ascertained with high precision in the original structural models. In other words, human structural analysis can overcome the uncertainty inherent in computational models, provided that these are sufficiently accurate to reliably identify interface residues.

With this approach we produced DV32.6 antibody mutants that could *a*) leave binding unaltered, proving that we can identify residues that are not critical for interaction despite belonging to the antigen binding loops or *b*) abolish binding to all serotypes. More importantly, whereas wild-type DV32.6 binds to all four DenV serotypes, we could design antibody mutants binding only to DenV2 or to DenV2 and DenV4. Increasing antibody specificity is valuable to eliminate unwanted cross-reactivity or to design bio-recognition elements. If an antibody has therapeutic purposes, however, one would seek to improve its neutralization properties, which would have a beneficial effect on dosage and therapy. It may be argued that if nature was not able to generate a better antibody, then there is no reason why we should be. However, individuals generate antibodies against a specific antigen that may be different from the target of antibody engineering. In the case of Dengue, for example, an antibody generated against DenV2 might be rendered more effective against DenV1. Since the individual from which the antibody was isolated had not been exposed to DenV1, the immune system would not have been able to optimize the antibody against this viral strain. Furthermore, a better antibody may exist in the serum of an immunized individual but it may simply fail to be successfully isolated.

As a proof of concept, we were able to engineer antibody mutants up to 40 times more effective than the starting molecule at neutralizing DenV. These results prove that even when high resolution x-ray information cannot be easily obtained, antibody/antigen complexes can be structurally characterized with sufficient accuracy to allow a fine control of their binding specificity and immunological properties. Although computational algorithms by themselves often fail to discriminate inaccurate solutions [18], by validating them with rapidly obtained NMR epitope mapping results we could achieve accuracy sufficient for rational mutagenesis. It may be argued that sophisticated NMR experiments could provide more information than the simple ^15^N-HSQC experiments used in our approach. However, the very simplicity of such experiments provides reasonably rapid results even in large, poorly behaved biological molecules where other NMR experiments may fail due to lack of sensitivity. In fact, even relatively low affinity complexes that tend to escape successful crystallization can be investigated by NMR mapping through HSQCs.

In addition to providing the basis for rational mutagenesis, analysis of the structure of the DV32.6/DIII complexes may be used to investigate the immunological properties of the antibody. DV32.6, in fact, is a poor neutralizer of DenV4 despite binding stronger to its epitope on DIII of DenV4 rather than DenV1 or DenV3 (KD is 34 nM for DenV4, 74 nM for DenV3 and 146 nM for DenV1). It may be argued that K_on_ or K_off_, rather than KD, might be more relevant for neutralization. However, there is no linear correlation between those and neutralization, either. K_off_ is better for DenV4 (5.1±1.3×10^−3^ s^−1^, 23.0±1.1×10^−3^ s^−1^ and 13.9±2.0×10^−3^ s^−1^ for DenV4, DenV3 and DenV1) and K_on_ is similar (1.5±0.1×10^−4^ nM^−1^s^−1^, 3.1±0.9×10^−4^ nM^−1^s^−1^ and 0.95±0.01×10^−4^ nM^−1^s^−1^ for DenV4, DenV3 and DenV1, respectively).

Given the same epitope and identical conditions, stronger binding should lead to stronger neutralization but this is not the case for DV32.6. One possibility, therefore, is that DenV4 has a different epitope from the other serotypes. NMR epitope mapping does not indicate this. The vast majority of the epitope residues are affected by antibody binding in all serotypes and the biological significance of those that aren’t is not evident. The different immunological profile is thus likely to arise from differences not evident at the epitope level that manifest themselves on the full viral particle, which the antibody needs to recognize in order to neutralize the virus.

DV32.6 recognizes a discontinuous epitope centered around the so-called A strand of DIII of all four Dengue serotypes. This region is close to the intermolecular interface in the E protein homodimers that form the surface of mature Dengue virus, with DIII from one E protein contacting DII of the other unit [30]. On the viral surface, the DV32.6 epitope is partly covered by DII residues. Antibodies 1A1D-2 [23] and 4E11 [24] were similarly shown to bind to partially inaccessible epitopes close to the DV32.6 binding site. Cryo-EM data shows that1A1D-2 binds to a virus conformation different from the one determined in the absence of antibodies. It is likely that DV32.6 sports a similar mechanism. In fact, steric clashes between DV32.6 and neighbouring E proteins are present when superimposing our models to the mature structure of the full virus, whereas no clash is detected when superimposing our models to the structure of the virus in complex with 1A1D-2. Presumably, structural dynamic of the viral surface exposes the epitope for a time sufficient for antibody binding. This theory is in agreement with ELISAs data on DV32.6 binding to the full virus: at 37°C there is sufficient dynamic movement to uncover the epitope and allow DV32.6 binding; at 4°C, instead, the dynamic process is slowed or altogether halted, the epitope is not made accessible for a sufficient amount of time and DV32.6 cannot bind effectively anymore to the virus.

We previously noted that antibody binding to DenV4 is likely to cause steric clashes on the viral surface, preventing efficient neutralization. Why is this not the case for the other serotypes? One possibility is that DV32.6 has less steric clashes on the virion of DenV1 to 3 and that relieving them requires less extensive, thus more likely, viral movements than DenV4. Another possibility is that different viral strains have different extent of dynamic movements on the viral surface. Indeed, antibody 2H12 was recently shown to bind a partly inaccessible DIII epitope [25], although not in the same exact region of DV32.6. Similarly to DV32.6, 2H12 has the same binding affinity for DIII of all serotypes (EC_50_ 0.4 nM in ELISA binding assays) but cannot neutralize DenV2. The authors, Midgley et al., suggest that the DenV2 virion might be less dynamic than other serotypes [25], resulting in less frequent exposure of the inaccessible antibody epitope and less efficient neutralization. In agreement with this interpretation, DV32.6 binds much stronger to DIII of DenV2 (KD 7 nM) than DenV1 (KD 145 nM), yet it neutralizes both strains equally well (EC50 is 1.8 µg/ml for DenV2 and 4.1 µg/ml for DenV1). The decreased binding affinity for DIII of DenV1 would be compensated by the additional rigidity of the virion of DenV2, resulting in similar neutralization. The same considerations hold true for the L-N27E antibody mutant, that neutralizes DenV2 and DenV3 equally well despite binding 100 times more strongly to DIII of DenV2 (KD 0.5 nM for DenV2 and 43 nM for DenV3, EC50 0.3 µg/ml in both cases).

However, in our case DenV4 would be the least dynamic virion, since the antibody binds strongly to DIII of this serotype but fails to neutralize the virus, suggesting that the epitope is seldom accessible on the viral surface. Midgley et al. do not describe a similar property for DenV4, although this serotype is less efficiently neutralized than DenV1 despite having similar binding affinity. One difference between the previously reported work and ours is that whereas both research teams use the same viral strains for DenV1 and DenV2, there are two aminoacid differences in DenV4 (L356F and N383D). Whether this is sufficient to justify a different viral flexibility is debatable.

Overall, the structural flexibility of Dengue viral strains remains largely unexplored. It is not clear, for instance, if it is exclusively an intrinsic property of the virion or if the antibodies can affect it. Antibody binding to one E protein on the viral surface, for instance, may distort the ordered structure of the virus and provoke a chain reaction making the epitopes on the other E proteins more readily accessible.

### Conclusions

Structural characterization of antibody/antigen complexes relies almost exclusively on x-ray information that may not always be available. Computational docking is emerging as an affordable alternative but it often fails to discriminate inaccurate solutions. We have shown that this problem can be alleviated by rejecting computational solutions that do not agree with a limited set of rapidly obtained experimental data. These may arise from viral escape mutants, peptide mapping or antigen mutagenesis; NMR epitope mapping, however, is a powerful tool for the detailed characterization of antibody binding sites with the potential to identify the epitope of several antibodies in a matter of hours, once assignment is available. We have shown that combining docking and NMR epitope mapping yields results sufficiently accurate for the rational modification of the antibody properties even in the absence of high resolution x-ray information. For antibody docking we have had good success with RosettaDock [21] but other programs like HADDOCK [31] are equally good. We believe that NMR validated computational docking can find widespread use in the structural characterization of antibody/antigen interactions, with implications for basic research, patenting purposes, screening and selection of candidate molecules as well as rational optimization of their binding and immunological properties.

## Materials and Methods

### Isolation of Human Monoclonal Antibody

Briefly, memory B cells were isolated from the blood serum of a donor recovered from DenV2 infection and immortalized with EBV as previously described [4]. The study protocol was approved by the Scientific and Ethical Committee of the Hospital for Tropical Diseases and the Oxford Tropical Research Ethical Committee. Written informed consent was provided. B cell clones were then tested for specificity by staining of Dengue infected cells and ELISA with recombinant E protein.

### Antigen and Antibodies Production and Purification

The sequence of DIII from each serotype was identical to that of the viral strains used for the binding and neutralization assays. DIII domains from E protein of each Dengue serotype were expressed in E. coli Rosetta cells with a pET21 vector (Novagen), induced at OD_600_ = 0.7 and harvested after 3 hours. After sonication and centrifugation, the pellet was repeatedly washed and centrifuged in sodium phosphate buffer pH 7.2, 1 M NaCl, 1 M urea and 1% Triton X-100. The pellet was finally resuspended in the same buffer with 8 M urea (buffer A). Following addition of 0.2% PEI and centrifugation, 65% ammonium sulphate was added to the supernatant. After centrifugation, the pellet was resuspended in buffer A and dialyzed for 3 days against 20 mM sodium phosphate buffer, 150 mM NaCl, 200 mM arginine, adding small amounts of pellet every 8 hours. DIIIs were finally purified on a superdex-75 size exclusion column (GE) with the buffer used for NMR and concentrated with Vivaspin concentrators (Sartorius). ^15^N and ^13^C labeled DIIIs, used for NMR experiments, were expressed in M9 minimal media using aptly labeled nutrients (NH_4_Cl and glucose) as sole source of nitrogen and carbon. DIIIs were proven to be correctly folded by NMR ^15^N-HSQC experiments.

DV32.6 from B-cell culture supernatant was purified by protein-A affinity chromatography followed by size-exclusion according to standard protocols. The F_ab_ fragments utilized for NMR experiments were obtained by enzymatic digestion with immobilized papain (Pierce) for 8 hours at 37°C, followed by dialysis, protein-A affinity and finally size exclusion chromatography against the NMR buffer. Recombinant wild-type antibody and mutants were expressed in HEK293T cells and purified as before. All samples were freshly prepared and mixed immediately before the required experiments.

### NMR Epitope Mapping

NMR epitope mapping experiments were conducted on the complex between DIII of each serotype and either full DV32.6 (1∶2 ratio to DIII) or its antigen binding fragment (F_ab_, 1∶1 ratio to DIII). Using the full antibody is more problematic for NMR due to its larger size but the enzymatic digestion required to obtain the F_ab_ fragments comported a significant loss of material and, thus, sample dilution. Use of F_ab_ fragments tended to provide better NMR results. TROSY versions or simple HSQCs experiments offered comparable sensitivity, they were both tested on each sample and the best one was chosen for the final experiment. Typical acquisition time was 30 minutes for free DIIIs and 18–24 hours for the complex at a temperature of 298 K. Typical concentration was around 0.25 mM in 20 mM NaPhosphate pH6, 50 mM NaCl. In a typical experiment, a ^15^N-HSQC spectrum of DIII from one serotype was recorded and a second spectrum was recorded after addition of the F_ab_ of DV32.6. The two spectra were visually compared and residues with different signal in the free and bound form were identified. Knowing the assignment, i.e. which protein residue corresponds to each NMR signal, DIII residues affected by DV32.6 binding were localized. NMR assignments of DIII from DenV1, DenV2 and DenV4 are publically available [12], [13], [14]. We assigned the backbone atoms of DIII of DenV3 with HNCACB and ^15^N-NOESY-HSQC experiments according to standard techniques. Spectra were recorded on Bruker 750 MHz and 800 MHz instruments equipped with cryoprobe and analyzed with the program Sparky [32].

### Antibody Modeling and Docking

DV32.6 was modelled according to the canonical structure method with the programs PIGS [19] and RosettaAntibody [33] as previously described [18]. 11 models with different H3 loop conformations were generated and independently used for docking. The structure of DIII from all serotypes is publically available [6], [13], [14], [17] and was used for docking.

Docking was performed using RosettaDock 2.3 as previously described [11]. In summary, a given antibody model was docked to DIII of one serotype; amongst the thousands of computationally generated complexes (typically 15,000), the one in better agreement with the NMR epitope mapping data was selected and further refined. If an antigen residue is in contact with the antibody in a docking decoy and is also affected by antibody binding in the NMR mapping experiment, then it is considered a valid contact. If it is not affected by antibody binding according to the NMR data then it is considered a violation. We select the docking decoys that maximize the number of valid contacts while avoiding violations. An antigen residue is defined “in contact” if either its N or H backbone atoms are within 7 Å of any antibody atom. This criterion proved to yield the most accurate results in extensive docking back-calculations of protein-protein complexes with known experimental structure and available NMR mapping information. By repeating the procedure for each of the 11 antibody starting models we obtained 11 putative complex structures. Not all of them equally satisfied the NMR epitope mapping data, so we finally retained only those complexes that had no significant disagreement with the experimental data and included all of them in the structural analysis. Computational models were discarded if the number of violations was greater than 1 or if the antibody showed extensive contacts with the antigen outside the canonical interaction regions (antigen binding loops and neighboring area). Amongst the remaining structures, those with a higher number of valid contacts were preferred. This was repeated for DIII of each Dengue serotypes. A table summarizing the docking and NMR epitope mapping results is offered as Table S1.

### Antibody Engineering

Antibody mutations were designed by visual analysis of the experimentally validated computational models. Single-point mutations were generated by site-directed mutagenesis of a vector encoding the wild-type antibody sequence using the QuikChange site-directed mutagenesis kit (Stratagene). The introduction of the desired mutations was confirmed by DNA sequencing. The PCR products were transformed into XL10-Blue supercompetent E. coli cells (Stratagene) and the plasmids purified according to standard techniques and transferred into HEK293T cells for antibody production.

### Binding and Neutralization Assays

All the neutralization and binding assays reported here were conducted on the wild-type antibody purified from immortalized B-cells isolated from the blood of a human donor, on the wild-type antibody cloned in HEK293T cells and on the mutated antibodies. Each measurement was conducted in duplicate, repeated 3 times and reported as the average of the resulting values. Results are normalized by considering as 100% the maximum point reached by the wild-type antibody, included as a reference in all experiments. EC_50_ were calculated as the midpoint transition in the s shaped curves after line fitting according to standard procedures.

Binding assays: 96-well ELISA plates were coated at 4°C with recombinant DIII or purified virus from DenV1-4. After washing and blocking, antibodies were added for 1 hr at 37°C. After further wash, bound antibodies were revealed using goat anti-human IgG coupled to alkaline phosphatase (Jackson Immunoresearch).

The ability of DV32.6 to neutralize the virus was assessed by flow cytometry assays measuring the number of VERO or RAJI cells infected by DenV vaccine strains in the presence of different amounts of antibody [4]. Different antibody concentrations were pre-incubated with DenV attenuated virus and used to infect VERO or RAJI cells. The percentage of infected cells was determined by fluorescence-activated cell sorting using standard procedures, staining the cells with mouse mAb 4G2 (ATCC, D1-4G2-4-15).

### Surface Plasmon Resonance Binding Assays

The affinity of DV32.6 for DIII of the four Dengue serotypes was determined at 25°C with a ProteOn XPR-36 instrument (Bio-Rad). The antibodies (concentration 150 nM) were immobilized on the surface of a GLM sensor chip through standard amine coupling. DIII was injected at a flow rate of 100 µl/min (contact time, 1 min) at different concentrations (300, 150, 50, 25 and 10 nM, running in parallel on separate channels); dissociation was followed for 15 min. Analytes responses were corrected for responses from buffer-only injection both on a channel with antibody immobilized and on a channel with no antibody immobilized. Curve fitting and data analysis were performed with Bio-Rad ProteOn Manager software (version 3.1.0.6).

## Supporting Information

Figure S1 **DV32.6 has a stronger binding affinity for its epitope on DIII of DenV4 than for the other serotypes (panel a) but it is less efficient at neutralizing it (panel c).** a) Binding assay (ELISA) for wt DV32.6 on DIII. The antibody concentration is on the x axis and increased y values correspond to increased binding. DIII from each Dengue serotype was immobilized on a surface in the presence of different amounts of antibodies as described in the methods. Binding appears stronger for DenV4 than other serotypes. The experiment was done in duplicate and repeated 3 times. b) Binding assay (ELISA) for wt DV32.6 on the full virus at 37°C. The purified virus from each serotype was immobilized on a surface in the presence of different amounts of antibodies. In contrast to the results of isolated DIII, binding to DenV4 is not stronger than to other serotypes. In fact, the binding curve does not reach plateaux in DenV4 at the tested antibody concentrations. The experiment was done in triplicate. c) Viral neutralization assay; the amount of infected cells (y axis) decreases at increasing antibody concentration (x axis). A higher amount of antibody is required to neutralize DenV4.
(TIF)

Figure S2 **^15^N HSQC spectra of DIII of the four Dengue serotypes free (blue) and in complex with antibody DV32.6 (red).** Residue V324 is affected by complex formation and shows chemical shift changes in DenV1, DenV3 and DenV4 but not DenV2.
(TIF)

Figure S3 **^15^N HSQC spectra of DIII of the four Dengue serotypes free (blue) and in complex with antibody DV32.6 (red).** Residue K310 shows chemical shift changes upon complex formation in DenV4 and DenV1 (smaller changes). The peak corresponding to the bound state disappears in DenV2 and DenV3, revealing that the residue is affected by antibody binding.
(TIF)

Figure S4 **Binding assay (ELISA) for all the antibody mutants designed to alter its properties in a predictable manner and mentioned in the main text.** The antibody concentration is on the x axis; increased y values correspond to increased binding. DIII from each Dengue serotype was immobilized on a surface in the presence of different amounts of antibodies as described.
(TIF)

Figure S5. (TIF)

Table S1 **NMR validation and docking results for the DV32.6/DIII complexes chosen as representative for each different antibody homology model used for docking (Model1 to Model10 and PIGS).** Models in agreement with the NMR epitope mapping data and chosen as final result for the structural analysis (see main text) are highlighted in green. If an antigen residue is in contact with the antibody in the computational model and is affected by complex formation in the NMR epitope mapping experiments then it is considered a “valid contact”. If it is in contact in the computational model but is not affected in the NMR epitope mapping experiment then it is considered a violation. The column “Valid-violations” indicates these numbers for each model. “Rosetta score” is the score calculated by the Rosetta-Dock scoring function, the lower the better; it is not sufficient by itself to discriminate incorrect models. “Rosetta ranking” indicates the rank, by score, assigned by Rosetta-Dock. A ranking of 1 means that the structure is the best scoring amongst the thousands of decoys generated in a typical docking run. Without NMR epitope mapping information one would have to rely entirely on the computational scoring function. In our case, instead, lower ranking models better satisfy the NMR epitope mapping information.
(PDF)
