# Supplementary figures and images for: Rational Engineering of a Human Anti-Dengue Antibody through Experimentally Validated Computational Docking
Source: PLoS One. 2013 Feb 6;8(2):e55561. doi: 10.1371/journal.pone.0055561 (PMC3566030; doi:10.1371/journal.pone.0055561)

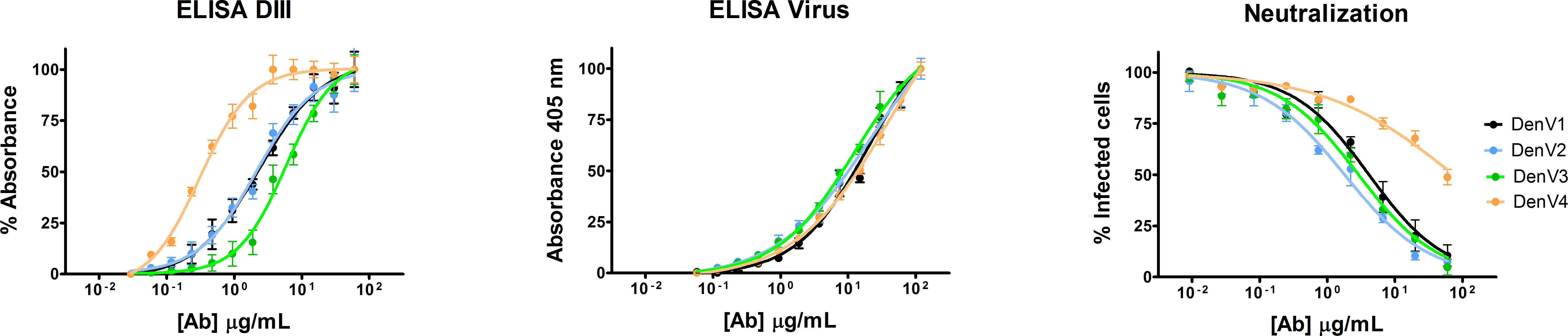

Supplement: Figure S1 — DV32.6 has a stronger binding affinity for its epitope on DIII of DenV4 than for the other serotypes (panel a) but it is less efficient at neutralizing it (panel c). a) Binding assay (ELISA) for wt DV32.6 on DIII. The antibody concentration is on the x axis and increased y values correspond to increased binding. DIII from each Dengue serotype was immobilized on a surface in the presence of different amounts of antibodies as described in the methods. Binding appears stronger for DenV4 than other serotypes. The experiment was done in duplicate and repeated 3 times. b) Binding assay (ELISA) for wt DV32.6 on the full virus at 37°C. The purified virus from each serotype was immobilized on a surface in the presence of different amounts of antibodies. In contrast to the results of isolated DIII, binding to DenV4 is not stronger than to other serotypes. In fact, the binding curve does not reach plateaux in DenV4 at the tested antibody concentrations. The experiment was done in triplicate. c) Viral neutralization assay; the amount of infected cells (y axis) decreases at increasing antibody concentration (x axis). A higher amount of antibody is required to neutralize DenV4. (TIF) [file pone.0055561.s001.tif]

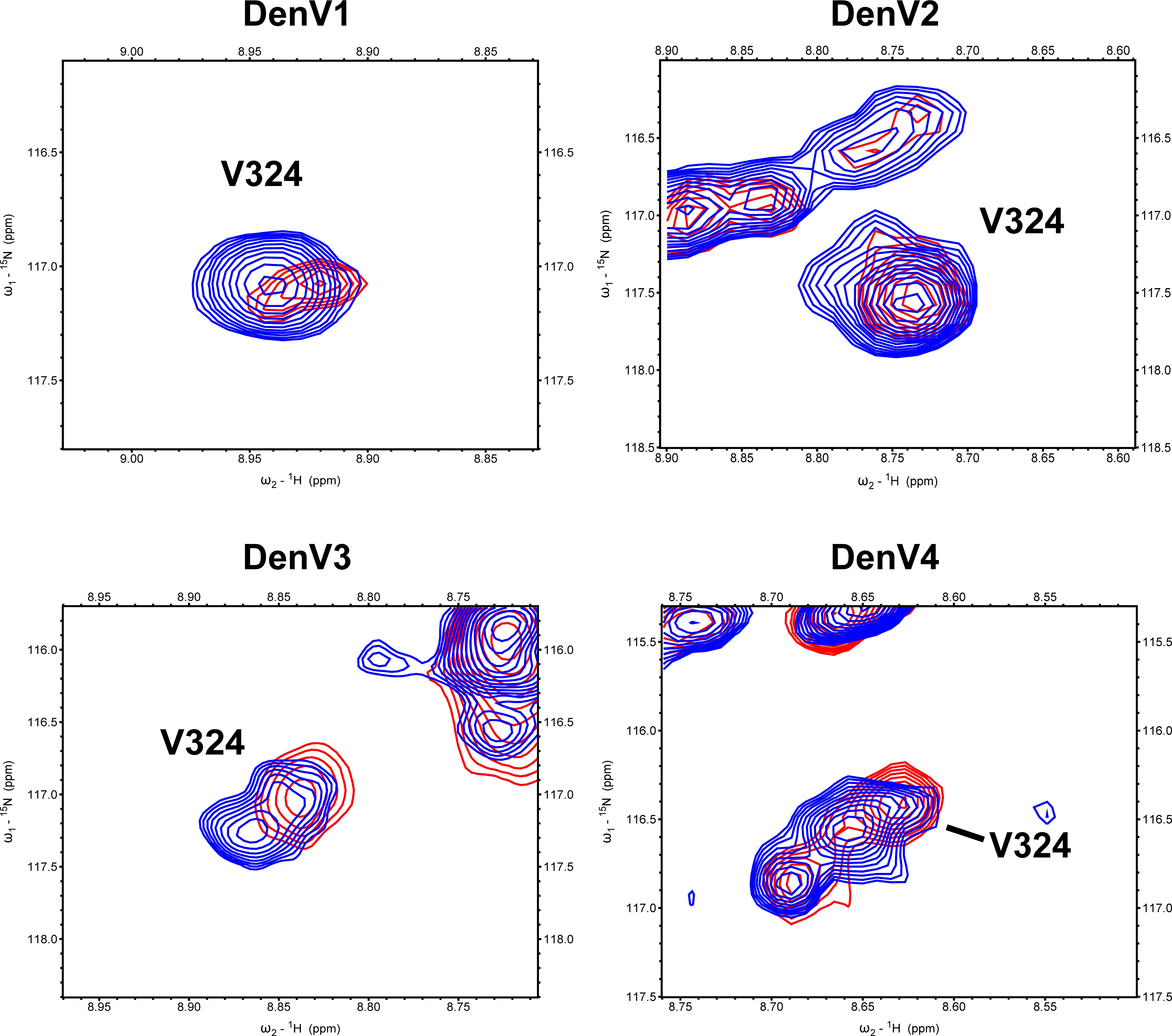

Supplement: Figure S2 — 15N HSQC spectra of DIII of the four Dengue serotypes free (blue) and in complex with antibody DV32.6 (red). Residue V324 is affected by complex formation and shows chemical shift changes in DenV1, DenV3 and DenV4 but not DenV2. (TIF) [file pone.0055561.s002.tif]

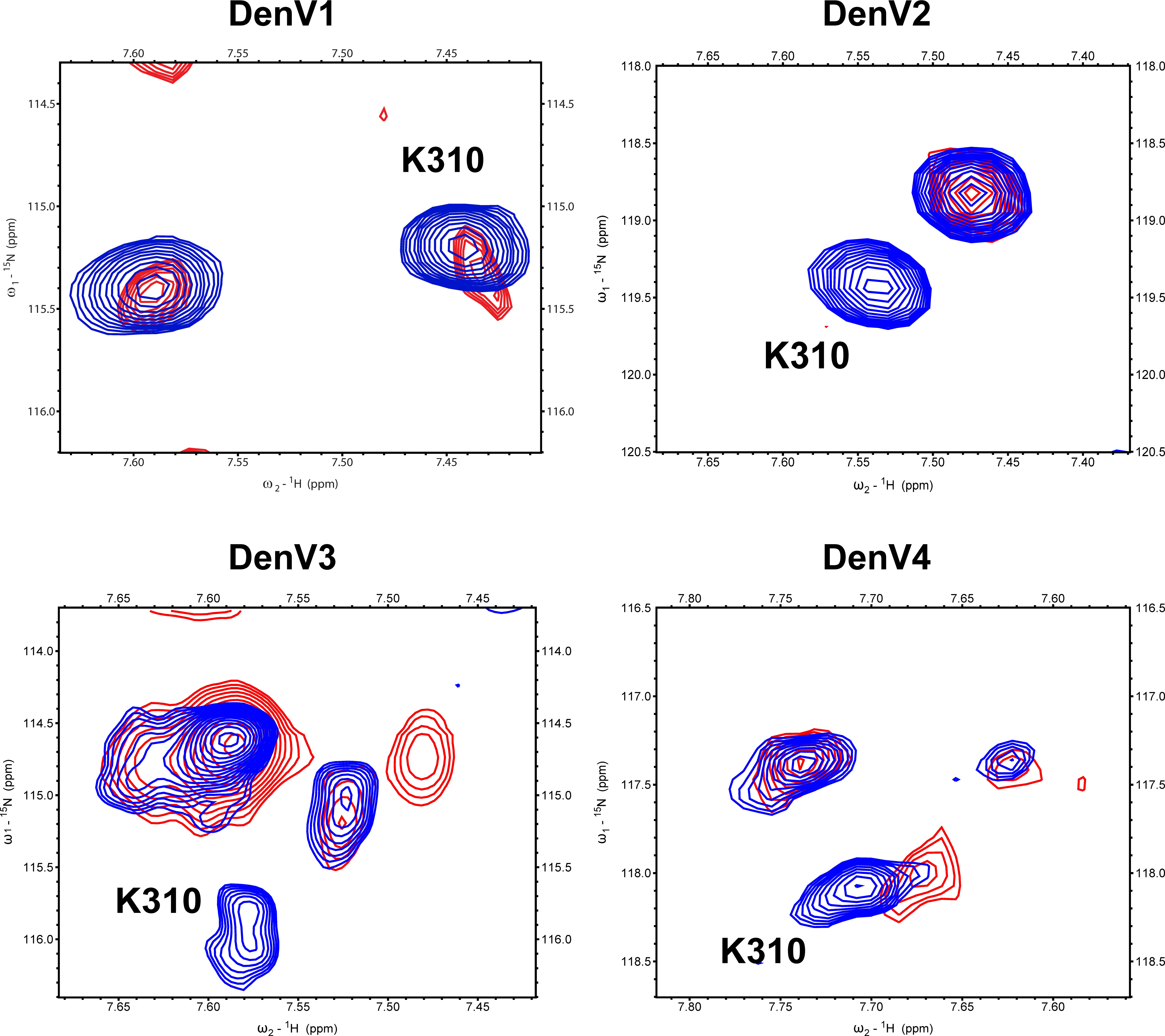

Supplement: Figure S3 — 15N HSQC spectra of DIII of the four Dengue serotypes free (blue) and in complex with antibody DV32.6 (red). Residue K310 shows chemical shift changes upon complex formation in DenV4 and DenV1 (smaller changes). The peak corresponding to the bound state disappears in DenV2 and DenV3, revealing that the residue is affected by antibody binding. (TIF) [file pone.0055561.s003.tif]

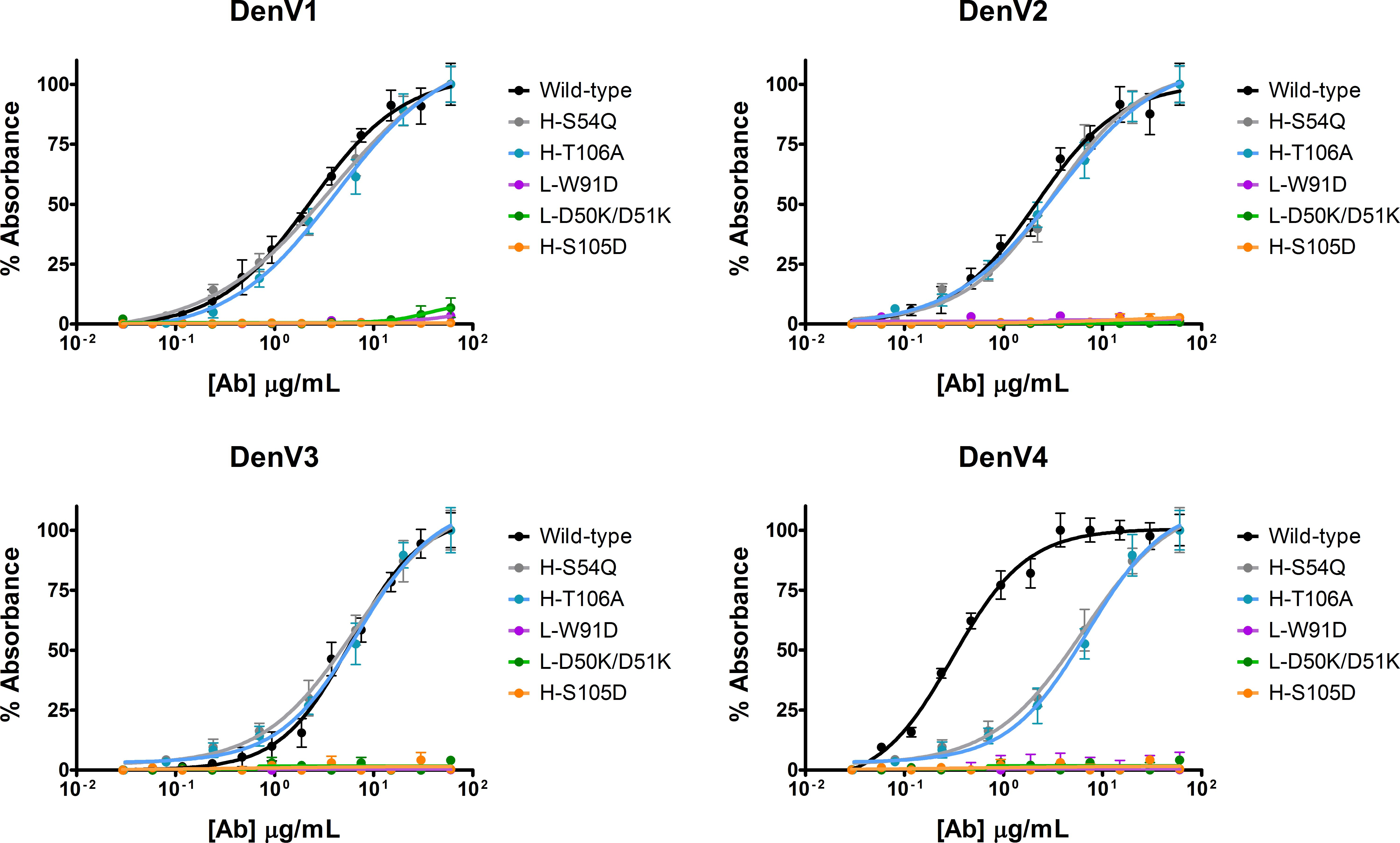

Supplement: Figure S4 — Binding assay (ELISA) for all the antibody mutants designed to alter its properties in a predictable manner and mentioned in the main text. The antibody concentration is on the x axis; increased y values correspond to increased binding. DIII from each Dengue serotype was immobilized on a surface in the presence of different amounts of antibodies as described. (TIF) [file pone.0055561.s004.tif]

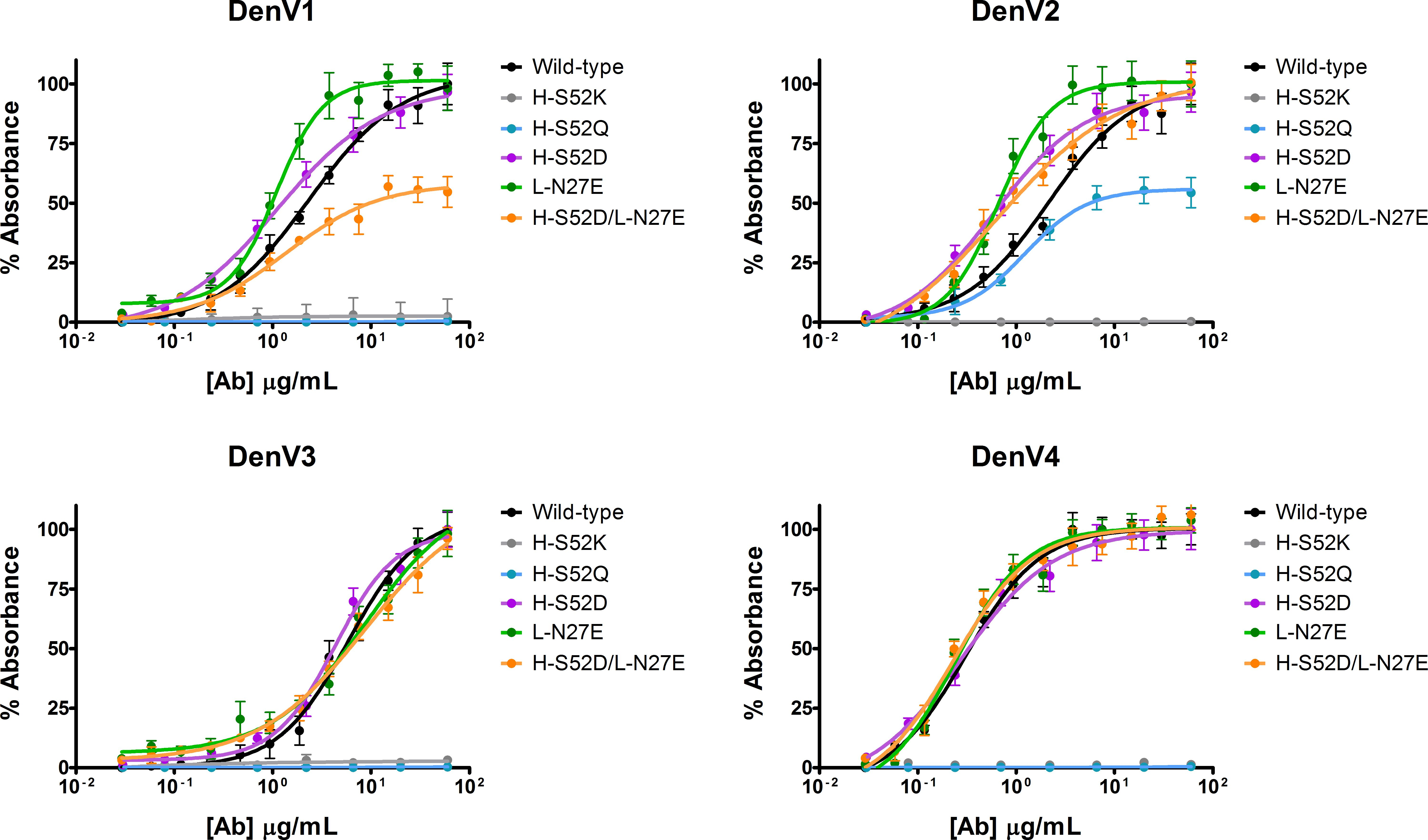

Supplement: Figure S5. — (TIF) [file pone.0055561.s005.tif]
