# Supplementary material for: Rational Engineering of a Human Anti-Dengue Antibody through Experimentally Validated Computational Docking
Source: PLoS One. 2013 Feb 6;8(2):e55561. doi: 10.1371/journal.pone.0055561 (PMC3566030; doi:10.1371/journal.pone.0055561)
Supplement: Table S1 — NMR validation and docking results for the DV32.6/DIII complexes chosen as representative for each different antibody homology model used for docking (Model1 to Model10 and PIGS). Models in agreement with the NMR epitope mapping data and chosen as final result for the structural analysis (see main text) are highlighted in green. If an antigen residue is in contact with the antibody in the computational model and is affected by complex formation in the NMR epitope mapping experiments then it is considered a “valid contact”. If it is in contact in the computational model but is not affected in the NMR epitope mapping experiment then it is considered a violation. The column “Valid-violations” indicates these numbers for each model. “Rosetta score” is the score calculated by the Rosetta-Dock scoring function, the lower the better; it is not sufficient by itself to discriminate incorrect models. “Rosetta ranking” indicates the rank, by score, assigned by Rosetta-Dock. A ranking of 1 means that the structure is the best scoring amongst the thousands of decoys generated in a typical docking run. Without NMR epitope mapping information one would have to rely entirely on the computational scoring function. In our case, instead, lower ranking models better satisfy the NMR epitope mapping information. (PDF) [file pone.0055561.s006.pdf]

DV32.6	DenV1 Valids-violations	DenV1 Rosetta score	DenV1 Rosetta Ranking
Model1	12-2	-227.98	12
Model2	18-2	-227.16	38
Model3	17-1	-227.1	31
Model4	17-0	-229.85	35
Model5	13-0	-229.61	45
Model6	12-4	-227.61	3
Model7	21-1	-228.76	4
Model8	18-0	-228.59	47
Model9	12-1	-227.27	5
Model10	10-1	-228.2	13
PIGS	15-4	-227.32	7

DenV2 Valids-violations	DenV2 Rosetta score	DenV2 Rosetta Ranking	DV32.6
11-1	-240.5	32	Model1
5-2	-239.45	86	Model2
12-1	-239.31	3	Model3
10-1	-244.08	1	Model4
12-1	-243.86	1	Model5
8-1	-239.52	2	Model6
12-2	-241.8	1	Model7
11-1	-242.42	1	Model8
13-1	-239.91	5	Model9
11-1	-241.12	1	Model10
6-2	-239.16	3	PIGS

DV32.6	DenV3 Valids-violations	DenV3 Rosetta score	DenV3 Rosetta Ranking
Model1	14-1	-233.04	4
Model2	13-2	-233.31	1
Model3	2-9	-232.49	4
Model4	1-4	-234.58	7
Model5	12-3	-234.06	61
Model6	14-2	-231.97	7
Model7	13-4	-232.56	15
Model8	11-1	-231.99	100
Model9	10-3	-230.21	173
Model10	9-0	-231.43	28
PIGS	13-5	-231.7	15

DenV4 Valids-violations	DenV4 Rosetta score	DenV4 Rosetta Ranking	DV32.6
8-0	-248.53	11	Model1
9-1	-250.4	4	Model2
10-1	-250.05	1	Model3
11-1	-253.11	1	Model4
11-0	-251.97	32	Model5
9-1	-249.08	11	Model6
9-0	-249.89	5	Model7
13-0	-251.31	8	Model8
12-0	-250.51	4	Model9
12-0	-249.77	4	Model10
7-3	-248.33	3	PIGS
